# Relationship between Hypoxic and Immune Pathways Activation in the Progression of Neuroinflammation: Role of HIF-1α and Th17 Cells

**DOI:** 10.3390/ijms24043073

**Published:** 2023-02-04

**Authors:** Consuelo Arias, Paulina Sepúlveda, Rodrigo L. Castillo, Luis A. Salazar

**Affiliations:** 1 Escuela de Kinesiología, Facultad de Odontología y Ciencias de la Rehabilitación, Universidad San Sebastián, Santiago 7500922, Chile; 2 Departamento de Ciencias Preclínicas, Facultad de Medicina, Universidad de La Frontera, Temuco 4811230, Chile; 3 Departamento de Medicina Interna Oriente, Facultad de Medicina, Universidad de Chile, Santiago 7500922, Chile; 4 Center of Molecular Biology and Pharmacogenetics, Department of Basic Sciences, Faculty of Medicine, Universidad de La Frontera, Temuco 4811230, Chile

**Keywords:** neuroinflammation, hypoxia, Th17, HIF-1α, autoimmune diseases

## Abstract

Neuroinflammation is a common event in degenerative diseases of the central and peripheral nervous system, triggered by alterations in the immune system or inflammatory cascade. The pathophysiology of these disorders is multifactorial, whereby the therapy available has low clinical efficacy. This review propounds the relationship between the deregulation of T helper cells and hypoxia, mainly Th17 and HIF-1α molecular pathways, events that are involved in the occurrence of the neuroinflammation. The clinical expression of neuroinflammation is included in prevalent pathologies such as multiple sclerosis, Guillain–Barré syndrome, and Alzheimer’s disease, among others. In addition, therapeutic targets are analyzed in relation to the pathways that induced neuroinflammation.

## 1. Introduction

Neuroinflammation is a complex immune response of neural tissue, to restrain infection and eliminate pathogens, cell debris, and mis-folded proteins in a generic manner; it involves all the cells present within the central nervous system (CNS), including the neurons, macroglia and microglia [1]. Emerging evidence suggests that neuroinflammation is a key player in many neurological disorders, including neurodegenerative diseases and CNS injury [2]. Both innate and adaptive immunity are involved in this process. Within this context, there are some factors, such as the dysregulated activation of the adaptative immune system, that can promote neuroinflammation [3,4], laying the foundations for different autoimmune diseases associated with our nervous system.

## 2. Cell Mediators: Helper T Cells Subpopulations

The adaptive immune system has a wide array of antigen receptors that can recognize a variety of pathogens [5,6,7]. In the case of T cells, once the specific antigens have been recognized by naive precursors in secondary lymphoid organs, cytokine-specific environmental signals are produced; these promote the differentiation of CD4+ effector T lymphocytes, such as T Helper cells (Th) or T regulatory (Treg) cells [8,9,10,11,12]. Th cells protect the body against pathogens cells and are essential to the innate immune system, as they can cooperate with and help B cells and cytotoxic T cells to enhance the protectiveness of T cells against a broad range of pathogenic microorganisms by releasing several types of cytokines in tissues [8]. On the other hand, Treg cells decrease the effector responses when they become hazardous to the host [9,10,11,12], inhibiting the proliferation of T cells and autoimmune responses, favoring immune tolerance [9,13,14].

The diverse subpopulations of Th cells perform different functions: Th1 cells are characterized by the secretion of interferon γ (IFN-γ) and tumor necrosis factor (TNF); they are the most common subset of memory effector T cells and offer protection against infection by bacteria, viruses, and intracellular parasites [8,11]. Th2 cells control extracellular parasites and have been linked to allergies [14,15]. Th9 cells are involved in the pathogenesis of allergies [16,17]. Th17 cells are responsible for fighting fungi and extracellular bacteria [9,12,15], and Th22 cells have an important role in cell proliferation, tissue regeneration, cellular defense, and inflammation [8,14].

## 3. Importance of the Treg/Th17 Relationship

Th17 cells are a subpopulation of CD4+ lymphocytes that have been linked to the Th1 cytokine profile and are characterized by their production of IL-17 and IL-22 [18,19].

Previous studies have found that IL-17 is a cytokine driving autoimmune and inflammatory diseases [20,21]. IL17 signals are received through the IL-17RA and IL-17RC receptor subunits, of which IL-17RA has a more ubiquitous expression and IL-17RC has a limited expression in mesenchymal and hematopoietic cells [20]. IL-17 triggers the activation of inflammatory transcription factors that induce the expression of NFKB and MAPKs pathways. Blocking IL17 in mice has been reported to reduce disease signs in several autoimmune model systems [20].

Th17 cells are not a “fixed” subset, since they are capable of converting into other lineage subsets influenced by the microenvironment [22]. Th17 cells phenotypically resemble differentiated memory T cells. The positive regulation of these cells has been related to the development and progression of chronic immune and inflammatory diseases, allergies and graft rejection reactions [23]. In fact, Th17 can induce autoimmune diseases such as collagen-induced arthritis, experimental autoimmune encephalomyelitis (EAE), intestinal inflammatory diseases, and others [9,24]. This phenotype is observed in the human microenvironments of cancer, autoimmune lesions and in organ transplantation [25,26].

Tregs is another lymphocyte subpopulation, characterized by the expression of high levels of CD25+, cytotoxic T-lymphocyte-associated protein 4 (CTLA-4) and the expression of transcription factor Foxp3. They have an essential role in anti-inflammatory, neurotrophic and neuroprotection functions [27], with a fundamental role in the maintenance of tolerance in the peripheral tissue and maintaining fetal-maternal tolerance [23]. These cells must know the antigens presented by the tolerogenic dendritic cells in an appropriate cytokine environment to proliferate, acquire functional maturity and exert immunosuppressive effects [23,28]; their deficiency or dysfunction have been linked to several inflammatory and autoimmune diseases, such as arthritis, irritable bowel syndrome, atopic dermatitis, psoriasis, and others [28].

Treg cells are capable of maintaining immune homeostasis and limiting inflammatory responses, for example, by inhibiting the Th17 response. This is how these two cell types play opposite roles during inflammatory and immune responses [18,29]. The balance of Th17 and Treg cells is crucial for immune homeostasis, and their imbalance plays a significant role in the inflammation reaction in autoimmune and neurodegenerative diseases [14].

### Regulation between Treg/Th17

The molecular pathways between Treg and Th17 are reciprocally interconnected [14]. Both the Th17 and Treg subpopulation requires the presence of TGF-β for their differentiation [30,31]. Virgin CD4+ T cells exposed to TGF-β co-express transcription factors specific to each line of Th17 and Treg: a retinoic acid receptor related to the orphan receptor γT (RORγT) in mice [32,33], known as RORc in humans [11,14], and the Foxp3, respectively. The expression of high levels of Foxp3 is necessary for the suppressive function of Treg. Foxp3 is negative dominant and antagonizes the function of RORγT [15,34]. This antagonism is annulled when there are other signals in the environment, such as IL-6 [9,35,36]. IL-6 has acquired an important role since it has been described as a key inflammatory mediator [37], facilitating the breakdown of the blood–brain barrier (BBB) and neuronal apoptosis, through an inflammation-dependent response and microglia-mediated downstream activation of the TLR4 and p-STAT3 pathway [38,39]. Therefore, the interaction between Foxp3, RORγT and signals such as IL-6, are the key elements that will determine which T cell phenotype will predominate [9,35,36].

**Figure 1:**
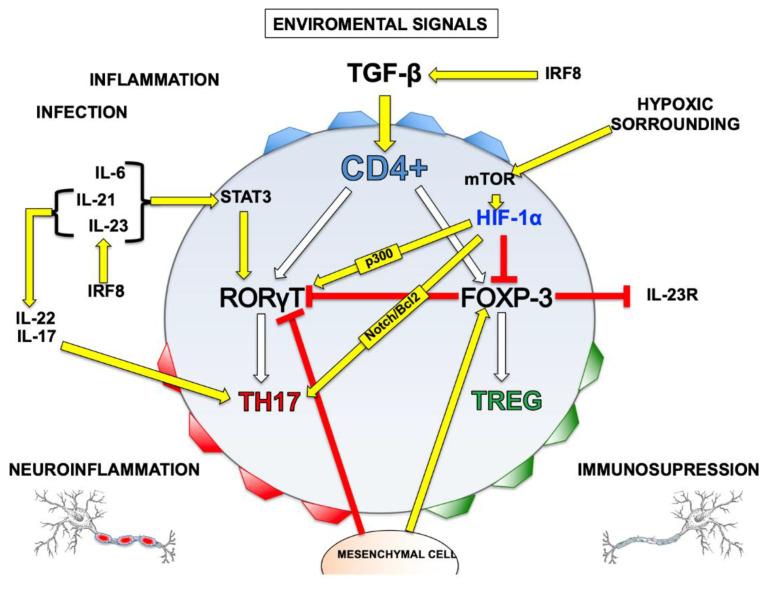
Signaling pathways in the regulation of Th17/Treg differentiation. Virgin CD4+ T cells exposed to TGF-β co-express transcription factors specific to each line of Th17 and Treg, RORγT and Foxp3, respectively. Foxp3 is dominant and antagonizes the function of RORγT. This antagonism is annulled when there are other signals, such as pro-inflammatory citocine favoring the differentiation of Th17 cells. HIF-1 promotes Th17 differentiation through the induction of RORγT and inhibits Treg differentiation through the degradation of the Foxp3. The p300 is a critical transcriptional cofactor for HIF-1. HIF1alfa/Notch/Bcl-2 signaling pathway controls the survival and apoptosis pattern in Th17. IL-6: Interleukin-6; IL-21: Interleukin-21; IL-23: Interleukin-23; IL-17: Interleukin-17; IL-23R: receptor de Interleukin-23; IRF8: Interferon regulatory factor 8; STAT3: Signal transducer and activator of transcription 3; TGF-β: Transforming growth factor beta; mTOR: mammalian target of rapamycin; HIF-1α: Hypoxia-inducible factor 1α; FOXP3: forkhead box P3; RORγT: RAR-related orphan receptor gamma; CD4+: Cluster differentiation 4; Treg: T regulator cells; TH17: T Helper cells 17.

The mechanism is explained as follows: IL-6, 21 and 23 activate the signal transducer and transcription activator 3 (Stat3), an element upstream of RORγT that induces its expression and promotes Th17 as a destination [9,15,40]. IL-21 and IL-23 maintain Th17 differentiation by increasing the IL-17 transcription, that is also another key specific to Th17 [9,15,41]. In addition, high TGF-β levels in the absence of IL-6 induce Foxp3 and repress the transcription of IL-23R. Foxp3 can be bonded to the RORγT protein and antagonize its bond to DNA by promoting the differentiation of Treg [9]. Recently it has been identified that the regulatory factor of interferon 8 (INF-8) activates the signaling of TGF-β, allowing the differentiation of Th17 cells, associated with higher levels of IL-23 [31,33]. It has also been described that mesenchymal stem cells inhibit the differentiation and function of Th17 cells, decreasing the number and activity of these cells at inflammation sites, an effect generated by negative regulation of the transcription factor RORγt and a positive regulation of Foxp3 [23,33] (Figure 1).

## 4. Hypoxia

Cell homeostasis, which can include oxygen homeostasis, is central to physiological processes such as growth, development, regulation of metabolic energy, angiogenesis and erythropoiesis, and for pathological processes such as anemia, ischemia, inflammation and cancer [42,43,44,45]. Tissue hypoxia arises when the oxygen levels in a tissue are lower than normal (normoxia). Hypoxia induces stress in organisms either through pathological or through nonpathological conditions [46]. Although hypoxia could have pathological consequences, such as a reduction in oxidative respiration, as well as reduction in the rate of electron transport in the mitochondria, increasing the generation of reactive oxygen species (ROS) [47], it also involves the maintenance of physiological functions such as angiogenesis or erythropoiesis [48].

Under hypoxic conditions, cells respond via the increase in hypoxia inducible factor (HIF) levels, which activate genes that modulate oxygen supply and consumption [46,49], and mediate the metabolic change from oxidative phosphorylation to anaerobic glycolysis, among others [9,50]. This transcription factor binds to specific DNA sequences that control the transcription of numerous genes in response to hypoxia [51]. Three types of HIF have been identified: 1, 2 and 3, with HIF-1 being the most studied [52,53]. HIF-1 is a heterodimeric transcription factor composed of an α subunit and a β subunit. Both subunits are members of the bHLH/PAS (basic helix–loop–helix/Per-Arnt-Sim homology) family of transcription factors, and both contain transactivation domains [52,54]. The α subunit expression is sensitive to oxygen, while the β subunit expression is constitutive [55].

HIF-1α and HIF-2α contain two transactivation domains, an oxygen-regulated C-terminal transactivation domain (CAD) and a more centrally located transactivation domain designated N-terminal transactivation domain (NAD). Both the NAD and CAD employ recruitment of the coactivators CBP/p300, SRC proto-oncogene, non-receptor tyrosine kinase (SRC-1), and transcription intermediary factor 2 (TIF-2) [54]. HIF-1α can be regulated by proteolytic degradation depending on the O2 levels exposure of the two domains. Under normoxic conditions, an enzyme that regulates HIF-1 stabilization, called prolyl hydroxylase (PHD), hydroxylates it, and then is recognized by the Von Hippel–Lindau (VHL) protein, ubiquitinated and degraded by a proteasome [51]. HIF stabilization is only a part of hypoxia-induced changes in the cells [56]. Nevertheless, HIF-1α can also be degraded independently of VHL and the existing oxygen pressure, through endoplasmic reticulum (ER) stress [57], hypoxia-associated factor (HAF) [58], SHARP1 [59], histone deacetylase inhibitor (HDACi) [60] and Parkin [61], among others.

### 4.1. Hypoxia and Th17 Relationship

Several studies have shown that HIF-1α is also involved in Th17/Treg regulation, being able to promote immunity mediated by Th17cells [25,26,62]. HIF-1α is required for Th17 differentiation, and its deficiency reduces the differentiation of this lineage [35]. The differentiation of virgin T cells to the Th17 lineage requires the positive regulation of genes involved in glycolysis, where HIF-1 fulfills a critical role in setting the metabolic state needed for Th17 development [10,13,15,46,62]. In fact, when naive T cells are cultivated under hypoxic conditions (5% O_2_, simulating physiological conditions), the differentiation of Th17 is increased, a phenomenon that requires HIF-1α and is induced by the activation of the mammalian target of rapamycin complex 1 (mTORC1) [10]. This differentiation appears to depend on mTORC1 downstream of PI3K–Akt axis [10,63], and also by a hypoxia-dependent and PI3K-Akt-independent pathway under reoxygenation conditions [10]. The mTORC1 activation promotes HIF-1 stimulation, contributing to the regulatory network of the Warburg effect in cancer progression [64], and regulates T cell metabolism and their differentiation into Th1 and Th17 subsets [65]. It has also been described that mTORC1 positively regulates IL17 expression through different pathways: STAT3, HIF-1α and S6K2 [66]. Moreover, in conditions where the differentiation of T lymphocytes to Th17 is stimulated (IL6 and TGF-β), there is a positive regulation of Stat3-dependent HIF-1α [50,55,62].

HIF-1 stimulates Th17 differentiation through RORγT induction and Treg differentiation inhibition, via an active process that promotes Foxp3 protein degradation, directly by means of ubiquitination [9,46]. Conversely, p300 is a critical transcriptional cofactor for HIF-1-mediated gene activation under hypoxia conditions [9,46,55], and a link has been identified between RORγT, HIF-1α and p300 and IL-17A, which promotes a permissive chromatin structure for its transcription [9,15,50] (Figure 1). Once HIF-1α and RORγT expression has reached a critical level, the cell is transformed into Th17, contributing to the inflammatory environment, which can increase hypoxia and produce a positive regulation of HIF-1α [50,62]. Therefore, in addition to participating in Th17 cell differentiation processes, HIF-1α activation also plays an important role in inflammatory and immune responses [67] (Figure 1).

Finally, it is suggested that another factor that could determine the memory Th17 cell fate would be the activation of the HIF-1α/Notch/Bcl-2 signaling pathway, which could control the survival and apoptosis of Th17 cells [25,68].

### 4.2. Hypoxia as Neuroglial Damage Mediator

All cells and tissues have the ability to respond to changes in oxygen levels [44,46]. The pathophysiological responses of damaged tissues against infectious, traumatic or inflammatory injury, are characterized by low glucose levels, high lactate levels and low pH levels and frequently by extreme degrees of hypoxia [43,55].

Brain tissue oxygen consumption is nearly 20% of the total oxygen consumed by the entire body. This event determined that this organ is found among the most sensitive tissues to hypoxia [69,70,71,72]. It has been found that hypoxia in the nervous system could have damaging or deleterious effects through inflammatory pathways, as well as neuroprotective effects via the adaptation that can be achieved and reported in chronic neurological conditions [72]. Sublethal hypoxia may improve an organism’s tolerance to subsequent hypoxia [73]. Exposure to moderate chronic hypoxia can increase life expectancy, and severe hypoxia can accelerate aging in animals models [72].

Some of the effects observed in hippocampal cells exposed to hypoxia are as follows: apoptotic nuclei formation, decrease in neuronal survival, increase in HIF-1α expression, loss of mitochondrial membrane potential, activation of caspase 3, and others [36]. In addition, in the CNS, hypoxia leads to a progressive increase in the levels of HIF-1α,vascular endothelial growth factor (VEGF) and Angiopoietin-2; all of these decrease its expression during prolonged exposure after the first week in hypoxia, in models of chronic hypobaric hypoxia [74]. This may be triggered by mechanisms of adaptation to hypoxia.

It has been described that there are other adaptive mechanisms to hypoxia, which are part of the Warburg shunt. Some can promote neuroprotection through a reduction in cytochrome C oxidase function and by an increase in the production of mitochondrial ROS, inducing a shift in energy gain towards astrocyte-mediated glycolysis [75]. Other metabolic changes can induce vulnerability, such as the pathological isoform switching of the glycolytic enzyme pyruvate kinase M (PKM) in induced neurons (iNs) from AD-patient-derived fibroblasts [76].

In addition, disruptions in energy supply, because of anoxia/ischemia, cause ionic homeostasis loss and the depolarization and rapid conduction failure of central fibers [43,71]. In the absence of an adequate energy supply, intra-axonal and extracellular calcium rises to toxic levels and irreversible axon damage occurs [77]. Indeed, disruption of calcium homeostasis following hypoxia, may alter synaptic plasticity and promote mitochondrial dysfunction, oxidative stress and apoptosis in the cerebral cortex, hippocampus and striatum, and contribute to the neurotoxicity of Aβ and the subsequent development of Alzheimer’s disease (AD) [45,78].

At the cellular level, hypoxia also generates the recruitment of microglia [79]. Microglia can express different but overlapping phenotypes, including the classical anti-inflammatory and the pro-inflammatory dual activated types. Therefore, the microglia-induced damage may depend on their phenotypic polarization after ischemia [80,81]. Activated microglia may have beneficial effects, phagocytosing cellular detritus and reducing inflammatory burst, previously reported by Neumann et al. [82] in a model of in vitro ischemia of hippocampal slices of rats. Hypoxia also could promote an inflammatory environment in the CNS tissue, which is exacerbated by the release of pro-inflammatory cytokines by glial cells [83,84].

Microglia activation causes the overproduction of inflammatory cytokines, which perpetuates hypoxic brain damage resulting in the death of neurons and oligodendrocytes, leukocyte infiltration, axonal degeneration and disruption of the BBB [46,70,85].

Although there may be a metabolic adaptation to acute and short-term hypoxia in some cells, sustained or severe hypoxia leads to increased production of ROS in the mitochondria, which contributes to cell death [86]. Oxidative stress has been linked to neuroinflammation and neuronal cell death, which are basis of several neurodegenerative diseases [87].

### 4.3. Hypoxia and Blood Brain Barrier Dysfunction

The pathogenic role of hypoxia, in triggering BBB anatomical and physiological disruption in neurological dysfunction, demonstrates an important protective role for vascular and glial integrity in the hypoxic brain [60].

The BBB is a dynamic barrier that represents the microvasculature of the CNS; it is composed of endothelial cells and mural cells (smooth muscle cells and pericytes), and has a basement membrane that separates into an inner and an outer sheet [88]. BBB also has microglial cells, astrocyte and perivascular macrophages [88,89]. Within the CNS blood vessels, the cells named above and T cells can interact, thereby regulating the properties of the BBB in response to infection or injury [88,89].

BBB’s integrity is essential for the normal physiology of the central nervous and glial system. Its disruption is associated with neurological pathologies that determine neurodegeneration events. This continuous non-fenestrated barrier allows endothelial cells to regulate CNS homeostasis and protect it against injury; meanwhile BBB dysfunction can trigger the entry of immune cells and molecules, leading to neuronal dysfunction and degeneration [88], and contributing to neurological and electrophysiological deficits [90]. The epithelial changes that can be generated are variable in the different types of pathology, but a fundamental piece in all of them is the dysfunction of the neurovascular unit (NVU) [91]. The cell populations that comprise the NVU change the profile of inflammatory responses against different injuries. In other cases, BBB alterations may be secondary to cerebrovascular abnormalities that create changes in cell permeability and epigenetic profiles, such as in neurodegenerative disease [92].

### 4.4. Hypoxia and Neuroinflammation

Neuroinflammation plays a relevant role in the development of different neurological diseases, through a response of the immune system to different noxious stimuli of the nervous system [93]. During neuroinflammation, cytokines and inflammatory mediators, such as chemokines and transforming growth factor–β (TGF-β), are released, affecting the BBB properties [94,95,96]. In this way, cytokines produced by pathogenic T cells, macrophages and brain microglia mediate a positive regulation of adhesion molecules at the BBB, with the subsequent influx of immune cells to the CNS [97]. The endothelial cells can release multiple inflammatory mediators and adhesion molecules, such as integrins (ICAM-1, VCAM-1) and E- and P-selectins [98]. These adhesion molecules allow myelin-reactive cells and inflammatory cells, such as granulocytes, neutrophils and macrophages, to penetrate the BBB under inflammatory conditions, which promotes an inflammatory cascade and the development of experimental autoimmune encephalomyelitis (EAE); this is a prerequisite for the for multiple sclerosis (MS) formation of lesions [32].

In the peripheral nervous system (PNS), neuroinflammation also continues, with the rupture of the blood-nerve barrier (BNB), accumulation of self-reactive T cells and macrophages due to chemotaxis and a progressive demyelination [99]. It has been observed that both Th17 and IL-17 cells mediate the disruption of the BBB [100] and BNB [101], by increasing the activation of matrix metalloproteinase-3 (MMP-3) and attracting neutrophils to the area of inflammation; this causes sustained axonal and myelin damage [32,98] through an increased inflammatory and autoimmune response [101]. In fact, Th17 cells have an important role in autoimmunity and inflammation of the CNS development during the early stages of EAE [98].

Hypoxia and inflammation are two events that coexist, share multiple connections and enhance each other. In addition, both are deregulated in a large number of diseases [102]. Hypoxia can induce inflammation and this development of inflammation is clinically relevant. On the other hand, inflammatory lesions frequently become severely hypoxic, through increased metabolic demands on cells or reduction in metabolic substrates [103]. However, studies on hypoxic environments shows that hypoxia itself represents an inflammatory stimulus, and it is the inflammation associated with the hypoxia that influences the prognosis of different organs exposed to ischemia [103,104]. The role of hypoxia in acute inflammation can be tissue-specific [105].

The adaptation to a hypoxic environment is also dependent on different inflammatory cellular and compounds induced through molecular mechanisms. Two recent mediators are described in cellular models of neurodegeneration, such as AD and PD, (i) metabolic adaptations and autophagy induction. Regarding the hypoxic reprogramming of metabolism, this is associated with the adaptation to excessive ROS production, and accompanies the mitochondrial changes in hypoxia [106]. In this case, hypoxia decreases the expression of glucose-6-phosphate dehydrogenase, thereby decreasing pentose phosphate pathway activity. This inevitably reduces the generation of nucleotides and cell proliferation. In addition, in animal models of intermittent hypoxia, the mitochondrial energetic regulation determines the impact of oxidative metabolism and ROS burst in neuroprotection. In this view, the brain is an extraordinarily plastic organ in which hypoxic stress initiates hormetic adaptations involving numerous molecular mediators, including HIFs and Nrf2, and myriad metabolic and enzymatic alterations, ultimately enhancing O_2_ supply, bioenergetics and cellular survival to preserve tissue integrity. Preclinical and clinical evidence clearly demonstrates that repeated moderate hypoxic bouts, i.e., chronic intermittent hypoxia, can preserve or enhance brain functions [72].

Under physiological conditions, autophagy is maintained at a low basal rate as part of quality control pathways to remove damaged proteins and organelles. However, it potently responds to external cellular microenvironments and can be influenced by nutrient and O_2_ availability to promote cell adaptation and survival [107]. BNIP3 (BCL2 Interacting Protein 3) emerged as a HIF-1α target. Accordingly, it is highly elevated in severe hypoxic conditions (~0.1–1% O_2_) in various cell lines and has pro-survival functions by mediating hypoxia-induced autophagy. Closely-related BNIP3L is also induced by hypoxia indicating both proteins are necessary for autophagy under these stressful circumstances. HIF-1α-dependent expression of BNIP3 has also been described as essential in mitophagy, as previously mentioned [42,108].

In the case of the brain, inflammation can affect it in different ways: (1) it can contribute to vascular wall atherosclerosis, causing vascular dementia and infarctions; (2) can compromise the BBB and its function, allowing leukocytes and antibodies to enter the brain; (3) brain produced antibodies can induce immune attacks, like those that occur in MS; (4) the inflammatory induced oedema; (5) some types of inflammation suppress neurogenesis; and (6) pathogens and protein can aggregate or damaged neurons can activate microglia and later kill neurons [99,109,110]. On the other side, the brain is highly vulnerable to intermittent hypoxia-related disorders, and this produces a different profile depending on the space and time characteristic of the hypoxia; this causes greater insult in the case of global and repetitive insults, and a less severe insult in hypoxias resulting from a single period of ischemia and of equivalent duration [111]. A large amount of neurotoxins are released into these environments as glutamate, TNF-α [99,109,110,112], IL-1β, binding Fas, cathepsin B and other proteases, contributing to neuronal loss or neurodegeneration [109,112]. Additionally, the activated inflammatory cells release large amounts of reactive oxygen species that further aggravate inflammation, exacerbating the neural insult due to the neuroinflammatory signals of microglia and peripheral macrophages [112].

### 4.5. Hypoxia and Autoimmune Diseases

Autoimmune diseases are characterized by immune systems’ loss of self-tolerance, which can be caused by environmental, genetic factors, or a combination of both [113,114]. Inflammation is a fundamental process in the activation of the immune system [115,116]. Deregulation of the immune system, due to the loss of immune tolerance or the presence of self-reactive T and B cells [116,117], can foster conditions associated with chronic inflammation, such as atherosclerosis [118].

To elucidate the role of microglia and other immune cells in neuronal damage, different genomic and transcriptomic assays have been performed in in vitro and in vivo models of AD [119]. For example, in brains of people with AD, T cells were associated with cerebral amyloid angiopathy blood vessels, and CD8+ T cells were specifically linked with microglia and amyloid plaque deposits [120]. Furthermore, it has been postulated that the systemic immune signals in AD originate outside the brain, therefore, its role in the pathogenesis of AD is not only limited to the brain [121].

Hypoxia influences our immune system through regulation of T cell differentiation, which is important not only in alloimmune regulation after transplantation, where the organ is exposed to severe hypoxic insults, but also in the modulation of autoimmune inflammation basis of different autoimmune diseases [43,49,122]. It has been reported that stabilization of HIF-1α promotes the survival and recruitment of neutrophils and induces phagocytes, which increases the microbicidal and proinflammatory capacities [15,24,50]. Stabilization of HIF-1 is directly bound to the disruption of the BBB, and its inhibition significantly improves the stability of the barrier. This hypoxia-mediated disruption is associated with several neurological diseases, such as cerebral infarction, brain trauma, autoimmune encephalitis, etc. [123]. It has also been observed that intermittent hypoxia (IH) increases the risk of developing or aggravating T-cell-mediated autoimmune diseases [124,125].

In this sense, IH is strongly associated with dementia via different mechanisms, including insulin resistance, inflammation, and ischemia. However, the direct effect of IH on the development or exacerbation of cognitive impairment remains unclear [126]. Recent evidence shows that the modulation of the intensity of hypoxia and the duration of the intermittence can determine neuronal and glial preconditioning phenomena. This is associated with biochemical phenomena, such as the reinforcement of antioxidant and anti-inflammatory mechanisms [127]. Regarding cognitive functions, it is probable that chronic IH determines apoptotic cell death and oxidative stress in the hippocampus. Administration of some antioxidants and neuropeptides might improve cognitive impairment induced by CIH, through inhibition of hippocampal apoptosis and oxidative stress [128]. A clinical pilot study suggested a potential utility of IH training (IHT) as a new non-pharmacological therapy, to improve cognitive function in pre-AD patients since IHT was able to generate improvement in cognitive test scores, along with a significant decrease in Aβ expression [129]. To further understand the complex relationship between IH and dementia, more molecular, clinical, and translational research in vitro and in vivo is required.

In addition, hypoxia facilitates differentiation of the proinflammatory Th17 phenotype, while simultaneously inhibiting the differentiation of tolerogenic Treg cells [50]. By contrast, the lack of HIF-1α results in a decrease in the development of Th17, increasing the differentiation of Treg cells and protecting the mouse from autoimmune neuroinflammation [35].

Th17 cells are important for the of the host’s mucosal defense and for mediating immunity against extracellular bacteria and fungi. However, deregulation of the Th17 response, characterized by overproduction of IL-17A and IL-17F, is particularly important as a cause or component in autoimmune diseases, and diseases associated with inflammation and destruction; these include rheumatoid arthritis, psoriasis, Parkinson, MS and Crohn’s disease [15,130,131,132]. In fact, it has been shown that Th17 cells can generate excitotoxicity in vitro in human neuronal culture [131,133], and IL-17 released by Th17 cells induces the production of other proinflammatory cytokines and chemokines, thereby promoting the recruitment of monocytes and neutrophils during the immune activation [133,134]. This phenomenon is associated with activation of the Janus kinase (JAK)-signal transducer and the activation of the transcription (STAT) signaling pathway [135]. In this sense, a higher frequency of Th17 has been detected in the blood of patients with PD and an increase in IL17 in the supernatant. These authors reported that blocking the IL17 signal prevents lymphocyte-induced neuronal death [132].

## 5. Autoimmune Diseases and Neuroinflammation: Role of HIF-1 and Th17

Identifying factors driven by autoimmunity in neurodegenerative diseases remains challenging [13,136]. Th17/Treg balance is regulated by different transcriptional factors and its imbalance is key in the development and progression of various diseases [137]. For example, in Treg cells from patients with RA, downregulation of STAT3 and HIF-1α has been described [137].

In the case of neuroinflammatory diseases, several studies have analyzed HIF-1 and Th17 related contribution [136]. Some studies indicate that hypoxia and HIF-1α promote FOXP3 transcription, favoring TH17 differentiation over Treg [102]. Moreover, mechanistic studies showed a Th17 cells increase, IL-17 production and an increase in the mobilization of T lymphocytes and macrophages that cross the vascular wall [138]. It is postulated that this regulation could be through the activation of the RORγt gene transcription; this recruits p300 and promotes the expression of TH17-associated genes and Th17 differentiation, together with a suppression in the differentiation of Treg via downregulation of Foxp3 protein [9].

Within the wide variety of diseases, we will analyze those that cause inflammatory lesions in the CNS and PNS through activation of the immune system, such as MS and inflammatory demyelinating disease, respectively, and inflammatory lesions neurodegenerative in nature, as with Alzheimer’s and Parkinson’s Disease [27,45,139].

### 5.1. Inflammatory Demyelinating Disease

Guillain–Barré syndrome (GBS) is an acute inflammatory disorder mediated by the immune system in the PNS, and characterized by inflammatory infiltration and damage to the myelin and axon [97,99]. Experimental autoimmune neuritis (EAN) is a model of this disease. EAN is mediated by T cells specific to a self-antigen, and is characterized as being self-limiting; it simulates many of the immunological and clinical characteristics of human acute inflammatory demyelinating polyradiculoneuropathy, a GBS subtype [101,140].

**Figure 2:**
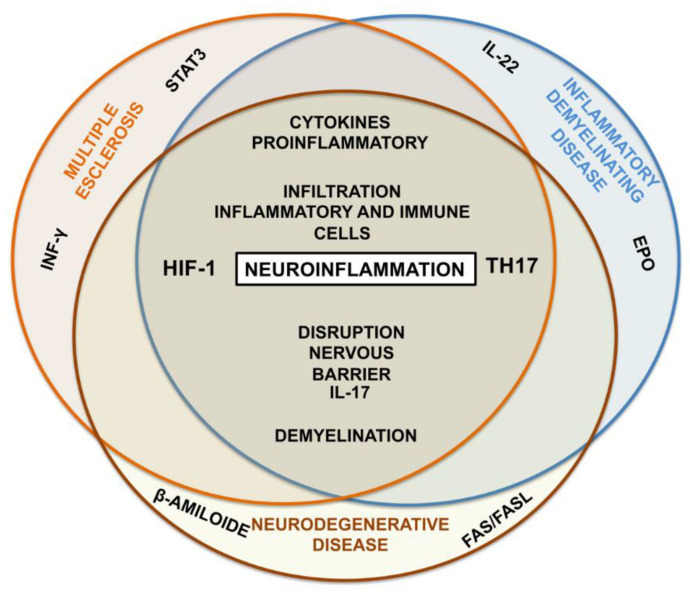
Diagram of the similarities observed in different diseases of the nervous system. The inflammatory demyelinating disease is characterized by inflammatory infiltration and damage to the myelin and axon. In the nerves of EAN, levels of mRNA and HIF-1α protein and erythropoietin are elevated. In addition, plasma IL-17A and IL-22 levels are remarkably elevated. Th17 cells contributing to the development of EAN and GBS. Multiple Sclerosis it is characterized by infiltration of Th17 cells, higher IL17 and IFN-γ serum cytokine levels and overexpression of IL-17 and HIF-1α. In addition, STAT3 is required for the development of this disease. Neurodegenerative disease involves accumulation of the beta amyloid protein and the activation of the Fas/FasL apoptotic pathway. HIF-1α, IL-17 and IL-22 expression is elevated and exists in infiltration of Th17 cells. This suggests that Th17 cells and the regulation of HIF-1α promote neuroinflammation in diseases of the Nervous System. IL-22: Interleukin-22; IL-17: Interleukin-17; STAT3: Signal transducer and activator of transcription 3; HIF-1α: Hypoxia-inducible factor 1α; TH17: T Helper cells 17; INF-γ: Interferon gamma; EPO: Erythropoietin.

Pathologically, EAN is characterized by the BNB rupture, accumulation of self-reactive T cells and macrophages in the PNS by chemotaxis and demyelination [99]. In the sciatic nerves of EAN, hypoxia and levels of mRNA and HIF-1α protein, HIF-2, erythropoietin (EPO) and the EPO receptor are induced in parallel at the peak of the disease and are reduced in periods of recovery, reflecting the hypoxic states of the peripheral nerves [101]. In addition, plasma IL-17A and IL-22 levels are remarkably elevated during the acute phase of GBS [99], and both the Th17 and IL-17A levels are associated with the severity of the disease [141]. Th17 cells mediate the inflammatory and autoimmune response in the human disease and the animal model [99], contributing to the development of EAN and GBS. Their differentiation after stimulation with the self-antigen is essential to determine the severity of the autoimmune disease [101] (Figure 2).

### 5.2. Multiple Sclerosis

Multiple sclerosis (MS) is a disease characterized by neuroinflammation in the CNS, and one of its animal models is EAE [98,125,142]. This model shows that both hypoxia and Th17 cells may play an important role in this disease [50,140].

Pathologically, in MS there is disruption of the BBB, inflammation [131,133] and infiltration of microglia/macrophages and T and B cells. It is characterized histologically by infiltration of encephalitogenic Th1/Th17 cells that favor activation of microglia and increase the autoimmune inflammation of the CNS [43,44], as well as an overexpression of IL-17 [114,142,143]. This disease, in many cases, continues with acute relapses; it includes progressive chronic neurological deterioration [98,122], beginning in young adult patients with intermittent episodes of neurological dysfunction, including visual alteration, ataxia, motor and sensorial deficit, and a loss of bowel and bladder control [32].

The neurological deficits and expression of HIF-1α have correlated quantitatively, temporally and spatially with the hypoxia generated in the gray and white matter in EAE, relating functional deficits to the neuroinflammation caused by tissue hypoxia [123]. Patients with nonparaneoplastic autoimmune encephalitis possess higher IL17 and IFN-γ serum cytokine levels than the controls. This reflects increased Th17 immunity, which could be involved in the pathogenesis of the disease [113]. It has also been observed that hypoxia facilitates the generation of Th17 in vitro, an important phenomenon for EAE since the absence of HIF-1α decreases the differentiation of Th17 in vivo, resulting in resistance to EAE. In addition, STAT3 is required for the differentiation of Th17 and mice with STAT3-deficient CD4+ T cells fail to regulate IL17 positively and to induce EAE [50]. In MS, there is an accumulation of inflammatory immune cells that migrate through the BBB; the chronic inflammation in the brain promotes the destruction of the myelin sheath, axonal damage, inappropriate activation of cells from the innate immune system and aberrant production of cytokines [98,135,143], which together, reduce the conduction velocity and cause a loss of function [143]. Ultimately, in EAE, a change in the differentiation of Th17 cells to Treg significantly improves the clinical symptoms [144] (Figure 2).

### 5.3. Alzheimer’s Disease

The etiological cause of AD involves accumulation of the beta amyloid protein, chronic inflammation reactions, oxidative stress, proteasome inhibition, and high cholesterol levels, where all these mechanisms are associated by a common factor: neuroinflammation [145,146]. In fact, it is suggested that chronic inflammation in the brain due to a deregulation of the innate immune system could be a precursory stage in the development of AD [123]. According to Zhang et al., Th17 cells are involved in the neurodegeneration produced in AD. They observed that, after injecting amyloid β1-42 (Aβ1-42) into the hippocampus of rats to induce AD, there was a disruption of the BBB, with the infiltration of Th17 cells in the cerebral parenchyma detected by an increase in the staining for the transcription factor RORγT; this was in addition to an increase in the expression of proinflammatory cytokines IL-17 and IL-22 [139,147]. This suggests that Th17 cells can infiltrate the cerebral parenchyma in AD, and could fulfill an important role in neuroinflammation and neurodegeneration by releasing proinflammatory cytokines, and by direct action through the Fas/FasL apoptotic pathway [45,148]. On the other hand, on AD patients, cerebral microvessels HIF-1α expression is elevated, compared to control patients [12] (Figure 2).

However, it has been mentioned that previous to amyloid accumulation, other alterations arise that could cause the onset of AD. This includes cerebral hypometabolism, triggered by events related to hypoxia or oxidative stress [149], or vascular changes similar to those that take place in vascular dementia induced by cerebral hypoxia, generated after a stroke, which increases the risk of developing AD [56].

## 6. Potential Therapeutic Targets

Due to the existing relation between Th17, HIF-1α and inflammation in neurodegenerative diseases, new therapeutic targets have been developed that could be used in the treatment of diseases with neuroinflammation.

The pathological niche created by hypoxia contributes to the development of inflammatory and autonomic diseases in a tissue- and context-dependent manner [105]. The energy crisis that results from hypoxia can predispose an organism to structural damage of the CNS, including demyelination and neuronal and axonal degeneration; when accompanied by immune alterations, these can be important components in several neurological disorders [123]. The selective dependence of Th17 differentiation in HIF-1α-mediated metabolic reprogramming provides a new target for the treatment of inflammatory and autoimmune diseases directed by Th17 [35,150]. As HIF-1 mediates processes adaptive to hypoxia, its inhibition could contribute to the prevention of cell damage as the disease progresses [53,96].

HIF-1 inhibitors, which increase the hydroxylation activity of PHD that may increase its degradation, could prevent the rupture of the BBB, associated with insult due to hypoxic damage and brain ischemia [53,96]. Inhibition of HIF-1α associated glycolysis could prevent neurodegenerative diseases [151], and protect the structure and permeability of the BBB; this could be through the suppression of HMGB1/TLR4/NF-κB signaling pathway-mediated neutrophil infiltration [152]. The problem with this treatment is that HIF1 has pleiotropic effects, and therefore the pharmacological manipulation of the HIF1 response must be careful to avoid side effects [43]. On the other hand, specific HIF2α inhibitors have been propounded, but their pharmacological effects are not fully known yet [105].

The increase in the Th17 response in the brain and peripheral tissues has a synergic effect on neuroinflammation and neurodegeneration in EAE, since the increase in IL17 and IL22 in the peripheral blood promotes the rupture of the BBB so that more Th17 cells migrate to the cerebral parenchyma, exacerbating the damage [139,148]. Therapies focused on inhibiting Th17 and their cytokines, and increasing Foxp3 expression in the early phases of EAN, could delay and suppress the clinical signs of the disease [141]. It has been observed that IFN-β inhibits the differentiation of human Th17 cells because it suppresses the production of Th17 cells; these also induce autoimmunity during the development of the disease and modulate the inflammation [32].

Because pro- and anti-inflammatory cytokines play an important role in the T cell immunomodulation, the reduction in proinflammatory cytokines and the increase in cytoprotective factors (such as antioxidants with a neuroprotective capacity) may be a potential therapeutic target [140,153]. The simultaneous inhibition of the multiple cytokines could break the cycle of inflammation characteristic of neuroinflammatory diseases [135]. For example, the inhibition of TNF induces IL-6 production with the resulting stimulation of the differentiation of T cells for Th17. Some of these drugs, such as infliximab or etanercept, have already been used in the treatment of rheumatoid arthritis and inflammatory bowel disorders [14]. PPAR-β/δ agonists would exert significant anti-inflammatory effects, and suppress the induction of chemokines and pro-inflammatory mediators (CXCL1, CXCL2, IL6, TNF-α), determine a reduction in neutrophil infiltration into the brain during ischemia, and protect against neuroinflammation [154]. In the case of their neuroprotective effects and PPAR-β/δ, agonists are tested as preventive strategies against AD and other neurodegenerative disorders, with favorable clinicals outcomes [155].

IL17-producing cells play a key role in promoting disease progression through amplification of the local immune response [156]. Some therapies involve monoclonal antibodies against IL-17 and the IL-17 receptor (IL17R). Combination of TNFα and IL17 inhibitors in human and mouse models of inflammatory diseases, show the most beneficial results [143]. For example, ixekizumab is a specific antibody for IL-17A, and brodalumab is an antibody in clinical development, the target of which is IL-17RA [14]. In addition, early intervention with IL17 neutralization in mouse models of Alzheimer’s disease may prevent early cognitive deficits, BBB disruption and synaptic disfunction [156].

The unregulated activation of the JAK/STAT signaling pathway has been implicated in several autoimmune and neurodegenerative diseases [157]. STAT3 signaling is a central component in the differentiation of cells to Th17 and Th17-dependent autoimmune processes; therefore, suppressing their activation is a promising treatment strategy [14,135,157]. AZD1480 is an ATP-competitive inhibitor of JAK1 and JAK2 that suppresses the activation of STAT, particularly STAT3, effectively suppressing the clinical symptoms of five preclinical models of MS. It reduces the Th17 response, the alterations in the function of dendritic cells and macrophages, decreases the infiltration of immune cells into the CNS, decreases demyelination, and suppresses the expression of proinflammatory chemokines and cytokines in vivo [135].

## 7. Concluding Remarks

Currently, neuroinflammation defines pathophysiological events, including several molecular pathways that regulate differentiation and maturation of the immune system. In fact, it is thought that the alteration of the metabolic environment in cells by hypoxia is what guides the activation of HIF, creating a feedback loop that connects the metabolism of immune cells with inflammation [105]. In this context, experimental and clinical evidence suggested that Th17 and HIF-1α are involved in the genesis of autoimmune and neurodegenerative diseases, contributing to the persistent pro-inflammatory state.

The pharmacological approach, which attenuates the response related to these molecular pathways, with mechanisms, for example, that sense hypoxia and its signaling pathway, could be used as a potential therapeutic target in the treatment of the disorders associated with neuroinflammation [103].

The different subpopulations of T lymphocytes and their plasticity under specific conditions, play a key role in neuroinflammatory diseases. For this reason, it is important to continue studying the contribution of each one, how they are able to change in the face of different stimuli, and how they trigger the onset or progression of the disease.

Finally, it is essential to determine the correct experimental model that would allow the study of these dynamic processes efficiently.
